# Atonic Dilatation of the Stomach

**Published:** 1902-12-06

**Authors:** 


### Hospital Clinics and Medical Progress.

### ATONIC DILATATION OF THE STOMACH.

Dealing with 100 cases of atonic dilatation of the stomach which he has seen during the last five years, Dr. Robert Saundby [1] considers the symptoms and treatment of this condition. First of all, however, it is necessary to define what is meant by the term. Such symptoms as splashing and visible peristalsis he does not regard as of serious value. They may be present when there is no dilatation and absent when that condition exists. Indeed, careful consideration of his cases has shown Dr. Saundby that the classical symptoms of dilated stomach were generally absent, and that the diagnosis would have been impossible by the usual methods. The method he employs consists in the administration of the stomach powders recommended by Dr. Eric Meinert, of Dresden, which contain 120 grains of sodium bicarbonate and 90 grains of tartaric acid, these quantities being dissolved separately in water and given successively to the patient. The administration of these powders rarely causes pain or distress, such discomfort as has been produced never having been more than trifling and temporary in character. The only needful warning is that the stomach should be empty at the time of their administration, otherwise vomiting of food may be caused; but if the stomach is empty, the most that occurs is the vomiting or eructation of inoffensive froth. It is to be noted that the administration of a stomach powder consisting of 15 to 30 grains of sodium bicarbonate with the same quantity of tartaric or citric acid, is altogether insufficient to distend the stomach properly or to define its shape, size, and outline. Nor is the inflation of the stomach by means of a syphon tube and the bellows of a spray producer a satisfactory proceeding. As to the causes to which the dilatation could be attributed, it appeared that in only 10 cases out of 100, namely those in which cancer or ulcer existed, could it be regarded as due to obstruction. Neurasthenia accounted for 29 and dyspepsia for 42, but no doubt many of the dyspeptic and other cases were neurasthenic. Pain was by far the most common symptom, and this was so in all the degrees of dilatation, and in both sexes. Vomiting came next, although this was not nearly so common as might have been expected. Loss of weight was noted in a good many cases, but in some it was definitely stated not to have occurred. It was met with proportionately more frequently, as was to be expected, among those cases which were due to obstruction—that is to cancer or ulcer—than in those due to atony. After pain and vomiting the most usual complaint made by the patients was of flatulence and water-brash. In regard to the chemistry of the stomach contents in these cases and the question of stomach motility the observations of Dr. Saundby are of much interest as showing the fallibility of certain signs on which much stress has been laid by some observers. Free hydrochloric was present in 32 out of 57 cases and absent in 20; in excess in two and deficient in three. Absence of hydrochloric acid is a point in favour of cancer, but it is too often present in chronic gastritis to make it absolutely trustworthy. Lactic acid was present in 48 cases and absent in 9, while butyric acid was present in only 4 out of the 57 cases in which the stomach contents were examined. Those cases also in which butyric acid was present did not differ essentially from others in which it was absent, either in the degree of dilatation or in the presence of obstruction. On examining the contents of the fasting stomach, sarcinæ were only seen in two, one of which was a case of cancer. Torulæ were slightly more frequent. In regard to stomach motility we may take it for granted that there must be delay in the discharge of its contents in these cases, and it is not of great practical importance to go any further. The general result of the examination of the fasting stomach in the morning is to show that it rarely contains anything but a few starch granules and epithelial cells.

Even when we have ascertained the presence of dilatation it has to be confessed that the prognosis in atonic cases depends much more on the duration of the symptoms and the co-existence of marked neurasthenia and bad general health than upon the extent of the dilatation as shown by distension with carbonic acid gas.

In regard to treatment it must be remembered that atonic dilatation is mostly a part of a neurasthenic condition. In cases which resist tonic treatment a modified Weir-Mitchell course is indispensable, including rest in bed for from three to six weeks, with general massage and perhaps abdominal massage and faradism, with the object of strengthening the abdominal walls. Every case upon going to bed is put upon an ounce of milk every hour diluted with barley water, soda water, or lime water, and no other food is allowed so long as pain continues; but if, as is usually the case, no pain is felt in the recumbent position, the milk is increased by 1 ounce hourly on successive days, until the maximum of 4 ounces per hour is reached. Should all go on well, 4 ounces of minced chicken are allowed twice a day with a very small slice of rusk or toast. Later comes minced mutton, with the addition of a little mashed potato and custard for dinner, and cocoa and rusk for breakfast instead of part of the milk. So little by little the patient is brought round to the normal diet of three or four meals a day, and when this result is attained he is allowed to get up and is either sent home or goes away for change of air and scene. In more obstinate cases however it may be necessary to wash out the stomach every night by means of the syphon tube. Evening is the best time, as it leaves the stomach clean for the night. If milk diet should fail, then recourse must be had to minced, lightly cooked mutton, with, if necessary, a small piece of toast or rusk to be eaten at the same time. Four oz. of the mutton, gradually increased to six oz., may be given three times a day, and this will agree with some patients with whom milk fails to produce improvement. For the constipation, which is often troublesome in these patients while they are lying in bed, a capsule of cascara may be given at bed time. If the tongue be foul one or two doses of calomel or blue pill may be given, with a bismuth, soda, and rhubarb mixture, or with one containing soda, rhubarb, cascara, nux vomica, ginger, and tincture of gentian, to be taken immediately before each meal. In cases of less severity, as measured by the duration of the malady, it may be sufficient to arrange for the patient to lie down for half an hour after each meal and to avoid fatigue, and in such cases a mixture of acid nitro-hydrochlor. dil. ♏ 15, with nux vomica, extract of cascara, ginger, and infusion of gentian, may be prescribed, to be taken immediately after each meal. Mild cases need not be dieted unless there are symptoms of gastritis, in which case fats, uncooked vegetable food of all kinds, coarse tea, and the stronger alcoholic drinks must be forbidden. But when there is no gastritis the patients may eat what they like. A light abdominal belt may be worn with advantage in some cases. In addition to what Dr. Saundby says as to the treatment which he advises, there is much that is of interest in his remarks about those forms of treatment which he does not recommend. For example, he does not place much dependence upon the various remedies which have been proposed with the object of assisting digestion, such as pepsine, pancreatin, and diastatic ferments. Again, as to surgery, the operation of gastro-jejunostomy appears a rational proceeding; but his experience of it in neurasthenic cases has not been favourable. In some cases the general condition has been worse after the operation than before. It is safer in incurable cases to provide a stomach tube, to be used with discretion.

[1] Brit. Med. Jour., Nov. 29.

